# Bio-Mimetic Sensors Based on Molecularly Imprinted Membranes

**DOI:** 10.3390/s140813863

**Published:** 2014-07-30

**Authors:** Catia Algieri, Enrico Drioli, Laura Guzzo, Laura Donato

**Affiliations:** Research Institute on Membrane Technology, ITM-CNR, c/o University of Calabria, Via P. Bucci, Cubo 17/C, 87030 Rende (CS), Italy; E-Mails: c.algieri@itm.cnr.it (C.A.); e.drioli@itm.cnr.it (E.D.); guzzolaura@alice.it (L.G.)

**Keywords:** molecular recognition, molecular imprinting, molecularly imprinted membranes, biosensors, bio-mimetic sensors

## Abstract

An important challenge for scientific research is the production of artificial systems able to mimic the recognition mechanisms occurring at the molecular level in living systems. A valid contribution in this direction resulted from the development of molecular imprinting. By means of this technology, selective molecular recognition sites are introduced in a polymer, thus conferring it bio-mimetic properties. The potential applications of these systems include affinity separations, medical diagnostics, drug delivery, catalysis, *etc.* Recently, bio-sensing systems using molecularly imprinted membranes, a special form of imprinted polymers, have received the attention of scientists in various fields. In these systems imprinted membranes are used as bio-mimetic recognition elements which are integrated with a transducer component. The direct and rapid determination of an interaction between the recognition element and the target analyte (template) was an encouraging factor for the development of such systems as alternatives to traditional bio-assay methods. Due to their high stability, sensitivity and specificity, bio-mimetic sensors-based membranes are used for environmental, food, and clinical uses. This review deals with the development of molecularly imprinted polymers and their different preparation methods. Referring to the last decades, the application of these membranes as bio-mimetic sensor devices will be also reported.

## 1. Introduction

Specific recognition at a molecular level represents a key necessity of living systems. Owing to this aspect, one of the most important challenges of many researchers is how to imitate the recognition ability of biological systems towards specific ligands. From this perspective, many efforts have been focused on the development of man-made materials with bio-mimetic properties. Among the different approaches used to produce these artificial systems, the “molecular imprinting technique” has received much attention in numerous fields due to its high selectivity for molecules of specific interest. The imprinting technology is based on the synthesis of a polymeric material endowed with specific recognition sites towards the molecule which must be recognized (template) [1]. This objective is accomplished by the addition of the template to a reaction mixture, which is constituted of a functional monomer, a cross-linking agent and a solvent. During the polymerization, the template is incorporated into the polymeric matrix and chemical groups of functional monomers will be arranged according to the shape and chemical properties of the template molecules. The extraction of the template from the obtained polymeric matrix will allow the formation of the template complementary recognition sites with high substrate selectivity and specificity [2,3]. In this way, selective detection and separation properties are introduced in the nascent polymer. In order to achieve efficient interactions between imprinted material and template, several aspects such as functional and shape complementarities, as well as the contributions due to the surrounding solvents have to be taken into account [4–6]. Imprinted materials are highly stable and useful for the development of new analytical methods that also work under relatively harsh operating conditions [7]. Up to now the mechanisms of recognition and the rules of template-monomer interactions are not well understood. Anyway, is logically possible to foresee how the change of one factor (e.g., the monomer/template/cross-linker ratio, polymerization method and temperature, *etc.*) has an effect on the recognition performance of such systems.

In the last decades the imprinting technique was employed to build up molecularly imprinted membranes (MIMs) able to selectively recognize target molecules from solutions by simple static adsorption or permeation through membrane devices [8]. A membrane is defined as a selective barrier interposed between two adjacent phases that regulates the transport of chemical species between them. Membrane technology had started to be developed a long time before of the birth of the imprinting technique and was in use in some research areas. Today it is one of the most developed technologies and it is widely used in numerous fields. The idea to introduce specific recognition sites into artificial membranes provided an unexpected impulse to the research activity focused on the development of new bio-mimetic recognition devices exhibiting high stability, sensitivity and specificity. In addition, compared to the traditional applications of imprinted polymers, MIMs can operate in a continuous way exploiting the features of membrane and molecular imprinting technologies [9]. In this context, Székely and co-workers [10], developed molecularly imprinted organic solvent nanofiltration membranes using polybenzimidazole (PBI) as functional polymer. Owing to the excellent chemical and solvent stability of PBI, the prepared membranes exhibited both shape-specific adsorption and size exclusion properties.

Different strategies were exploited for the development of MIMs in both, flat-sheet and hollow fiber configurations. Flat-sheet membranes have a paper-like configuration. They are thin films with porous or dense structures. Hollow fiber membranes have a long spaghetti-like tube configuration. Their diameter varies over a wide range from to 50 to 3000 micrometers. Fibers can have a macroporopus structure with a dense selective layer on the outside surface. As examples, Wang *et al.* prepared flat-sheet MIMs for the specific recognition of curcumin using the photo-copolymerization method [11]. In another approach, the phase inversion technique was applied for the development of flat-sheet MIMs for the recognition of Rhodamine B [12] and uric acid [13]. Hollow fiber composite membranes were also prepared *via* thermal co-polymerization. The antibiotic levofloxacin was used as template [14].

Using the product of an enzyme reaction as template an imprinted membrane can be utilized for assisting in synthetic reactions by continuously removing the product from the bulk solution by complexation. In a similar manner, MIMs can be used for recovering chemical substances coming from fermentation broths such as antibiotics and organic acids, as well in chiral resolution of enantiomers, substances utilized in pharmaceutical field.

Recently, bio-sensing systems using MIMs have received much attention from scientists in various areas. Biosensors are tools of molecular dimensions that are engineered for sensing different analytes on the basis of bio-specific recognition enabling the detection and quantification in a single operation step. In a sensor a MIM is used as bio-mimetic recognition element which is integrated with a transducer component. The detection is accomplished on the base of specific characteristics of the target analyte (such as its IR spectrum/fluorescence/electrochemical activity) or modification of physico-chemical parameters of the system in response to the binding with the template. A chemical or physical signal is generated upon the binding of the template to the membrane, which is translated into a quantifiable output signal by transducer. The direct and rapid determination of an interaction between the recognition element and the template represents an encouraging factor for the development of such systems in alternative to the traditional methods of bio-assay. This is because they do not need the addition of secondary reagent and the separation of free and bound reactants [15]. Bio-mimetic sensors-based membranes have been developed for environmental, food, and clinical uses. This review deals with the different preparation methods of molecularly imprinted membranes. Referring to the last decades, the application of these membranes as bio-mimetic sensor devices will be also reported.

## 2. Molecularly Imprinted Polymers

Although the bio-molecules used as sensing elements in molecular recognition processes are highly specific and sensitive, they suffer the disadvantages of being fragile and expensive. In addition, they possess low density of recognition sites; their regeneration as well as their storage is not easy and restricted. The technique of molecular imprinting is an advantageous alternative to surmount problems linked with bio-molecules such as enzymes, antibodies, cells, animal or plant tissues. In fact, imprinted materials can be synthesized in a short time, are robust and highly thermostable. They can be also used in a wide range of solvents, pH and metal ion solutions with different ionic strength. Furthermore, they are easily regenerated and stored at room temperature without loss of efficiency. In this perspective, new polymeric receptors may be designed towards a wide variety of target analytes over traditional biological receptors [16–18]. The concept of specific recognition was pioneered in 1955 by Dickey who prepared “specific adsorbents” of silica gel by simply acidifying commercial silicate solutions containing methyl orange or one of its homologs [19,20]. However, the imprinting on inorganic silica was not quite popular due to the lack of repeatability in materials preparation and their low selective properties. In 1972, Wulff and Sarhan [21] and Klots and Takagishi [22], in parallel, reported the first examples of molecular imprinting of organic network polymers.

To produce molecularly imprinted polymers (MIPs) three essential elements are required: (1) the *target molecule* (or *template*) which is the substance that will be imprinted in the polymer; (2) the *functional monomer* which is a compound having chemical and shape complementarity to the template and will polymerize in order to form polymer matrix; (3) the *cross-linking agent* (or *cross-linker*) which is a multifunctional molecule containing two or more reactive split ends able to interact via chemical bound with specific functional groups present on other chemical substances (e.g., linear polymer chains).

**Scheme 1.**
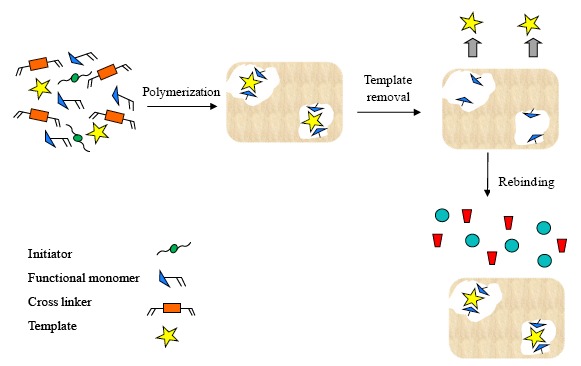
Representation of the molecular imprinting process.

The synthesis entails the following steps: (1) the template molecules form pre-polymerization complexes with polymerizable functional monomers capable of interacting with them. In this phase, the functional monomers arrange around the template in order to create the recognition sites. (2) The pre-formed complexes are polymerized with the cross-linker molecules into a rigid polymer to maintain the location of the functional groups for binding the template. (3) After the polymerization step, the template is removed from the polymeric matrix allowing to generate the specific recognition sites which possess the molecular memory of the template. In fact, they are capable to selectively rebind the template molecules with respect other compounds, including their structural analogues. In Scheme 1, a representation of the molecular imprinting process is reported.

The heart of the process is the template, which should be chemically inert under the polymerization conditions *i.e.*, polymerization temperature, UV irradiation, *etc.* [6]. The polymerization is started by the addition of an initiator to the pre-polymerization mixture constituted of the three abovementioned elements. An appropriate solvent (porogen) is used to dissolve them. The nature and volume of solvent are important to control the morphology and the pore volume of the polymer [23,24]. In the high diluted reaction environment, the growing polymer is not able to live in the entire available volume and more small polymer particles/powders having higher surface area (and more accessible recognition sites) are obtained. The final structure and performance of the polymer depends also on other factors such as the selection of appropriate functional monomer and cross-linker. Their concentration is also critical for ensuring the interactions with the reactive groups of the template [6,25–34]. To exploit the complex formation and thus the imprinting effect it is extremely important to combine in a complementary way the functionality of the template with that of the functional monomer. As a rule, this latter is used in excess in relation to the total number of target molecules. In addition, if two or more functional monomers are simultaneously used in a co-polymerization is important to keep in mind their reactivity ratio in order to guarantee the feasibility of the synthesis.

In general, the functional monomer possesses two functional groups. At one end it forms non-covalent or reversible covalent bonds with the template. At the other end of the molecule there is a chemical group covalently interacting with the cross-linker or other monomers [16]. The presence of the cross-linker confers sufficient rigidity to the synthesized polymer, which is necessary for the formation and stabilization of the specific recognition sites. In fact, the cross-linker freezes the polymeric network in a suitable configuration to interact with the template. Furthermore, in addition to the solvent, the cross-linker is important in controlling the morphology of the polymer matrix (whether it is gel-type, macroporous or a microgel powder) [6]. To be able to generate materials with adequate mechanical stability, high cross-linker/functional monomer ratios are usually used. Yungerman and Srebnik [35] investigated the imprinting quality of cross-linked polymer networks using molecular dynamics simulations. The authors carried out a topological analysis of the imprinted network configuration before and after template removal. They concluded that the formation of distinct individual cavities retaining the size and shape of the template is enhanced by high degrees of cross-linking and low template concentrations. This is because of the reduction of the templates molecules aggregation in the pre-polymerization mixture and the deformation of the recognition sites (due to relaxation of the polymer network once the template has been removed). Polymers with cross-linker molar ratios in excess of 80% with respect to the monomer are usually used to obtain materials with adequate mechanical stability and good recognition performance [36]. However, an excessive cross-linker ratio will result in an extreme polymer rigidity, which adversely affects the interactions between the polymeric matrix and the template [26,37,38]. In addition, the number of recognition sites per unit mass of polymer are reduced [6,39]. On the other hand, using an insufficient cross-linker ratio, the polymer cannot maintain stable the configuration of the recognition sites because of the degree of low cross-linking [39].

Another important aspect to be taken into account to produce imprinted materials exhibiting good performance is the polymerization temperature. In fact, this parameter also affects the polymerization process (e.g., reaction completeness and reaction rate) and polymer structure (e.g., pore structure and swelling properties), which in turn influence the quality and quantity of recognition sites. Normally, lower temperatures will stabilize the template–functional monomers complexes. In 1988, Sellergren *et al.* demonstrated that a decrease of the polymerization temperature allowed one to increase the concentration of the pre-polymerization complex and consequently the number of recognition sites (creating during polymerization) compared with the same polymer synthesized at higher temperature [40]. Other synthesis, performed in a temperature range from 0 °C to 60 °C, using different initiators, demonstrated that polymers produced at low temperature exhibited better specificity with respect to the polymers synthesized at higher temperature [41,42]. On the other hand, a weak point of the polymerization performed at low temperature is the necessity of a longer time for the completion of the reaction [43,44]. In addition, when radical polymerization is used, another important parameter to be taken into account is the initiator system. In fact, when conventional initiators (including peroxides, azo compounds and thermal iniferters) are used a high polymerization temperature is typically required to guarantee the rapid decomposition of initiator avoiding the formation of toxic chemical side products [45]. In 2010 Cirillo *et al.* [46] performed polymerization processes at lower temperatures by using Fenton reagents as the redox initiator system. The advantage of this kind of initiator system were the low work temperature, the short reaction time (2 h for this synsthesis *vs* 24 h for the synthesis of conventional MIP synthesized by azo-initiators) and the absence of any kind of toxic reaction products [46]. In addition to those already mentioned, other interesting papers have discussed the influence of the polymerization conditions on the performance of MIPs [47–51].

Two main types of chemical interactions can be used in the preparation of molecularly imprinted materials: covalent and non-covalent binding [27,52,53]. The former, pioneered by Wulff [21,54–56], is based on a reversible covalent attachment strategy between the template and the functional monomer in the pre-polymerization mixture. After polymerization the bonds are chemically cleaved in order to set free the template and remove it. The interactions template/functional monomer takes place via the formation of covalent bonds also in the rebinding action. The second type of interaction, proposed by Mosbach and Sellergren [57–59], is based on non-covalent self-assembly (*i.e.*, hydrogen, hydrophobic and Van der Waals interactions, metal chelation, *etc.*) of the template with functional monomers to position them in a precise spatial arrangement prior to polymerization. Subsequent free radical polymerization with the cross-linking agent allows one to obtain an imprinted polymeric network.

Like all the things, both synthetic strategies possess advantages and disadvantages. Owing to the possibility to control the stoichiometry of the imprint materials, the covalent approach allows obtaining more homogeneous recognition sites than the non-covalent one. Covalent-imprinted polymers are very stable and selective. To their disadvantage, they have a limited number of functional groups to interact with target molecules and exhibit slow binding kinetics. In addition, their repetitive use in cleaving and rebinding templates can be difficult, because of the restricted interactions. Finally, the covalent interaction is different from the natural recognition at molecular level that occurs in biological systems.

The non-covalent imprinting approach had the advantage that the monomer/template complex can be formed by self-assembly simply by mixing the interacting molecules, yet, the template is removed under mild conditions and a high number of recognition sites may be generated. A weak point of this method is that the template-functional monomer interactions are less strong than covalent bonds. In addition, in this approach, the interactions are governed by an equilibrium process and therefore the functional monomers are used in excess with respect to the template to move the equilibrium versus the formation of the template-monomer complexes. As a consequence, the excess of free monomer is randomly distributed in the polymeric matrix allowing one to also generate non-homogeneous and/or non-selective recognition sites [47,60,61].

Sometimes, polymers were synthesized by means a combination of covalent and non-covalent imprinting to surmount the problems related to the independent application of each method. In this case, the monomers and the print molecules, were covalently joined during the polymerization and the subsequent rebinding took place by non-covalent interactions. This approach is called the sacrificial spacer method and was introduced by Whitcombe [62].

At present, the most used technique to produce imprinted materials is non-covalent imprinting. This route offers some advantages over the covalent approach: it is easy dissolve monomer and template in a solvent and there exist a variety of monomers having chemical functionalities able to interact with any desired template molecule [29]. Besides, the removal of the template is easier with respect to the covalent binding approach. In fact, non-covalent interactions like hydrogen bonding are reversed by washing with methanol, methanol/acetic acid, or in aqueous solutions of an acid or a base. This wash allows removal of the template from the polymeric network after the polymerization process [28]. Finally, non-covalent interactions characterize the basis of the molecular recognition in living systems. In effect, like non-covalent molecularly imprinted materials, biological receptors such as antibodies and enzymes, recognize their target molecule via complementary ionic, hydrogen-bonding and hydrophobic interactions.

However, the removal of the template is a critical step in the preparation of imprinted polymers. In fact, like in the case of non-covalent binding, the interactions between polymer and template can sometimes make hard the removal of the last traces of template, even after washing the material many times. This is a problem which may leads to the bleeding of the residual template when the polymer is used [45,63]. In addition, it must be take into account that if there are residual template molecules in the imprinted matrix, less recognition sites will be available for rebinding, thus lowering the polymer efficiency [64]. Some researchers have investigated the possibility of achieving complete template removal. In 2001, Ellwanger *et al.* [65] employed different post-polymerization treatments (thermal annealing, Soxlet extraction, microwave-assisted extraction and supercritical fluid template desorption) aiming to a complete removal of clenbuterol and and L-phenylalanine anilide from their respective imprinted polymers. Referring to clenbuterol, the lowest bleeding level was observed after microwave-assisted extraction. In the case of the polymer imprinted with L-phenylalanine anilide, the lowest bleeding was found after extensive on-line washing in solvents containing acid or base additives and after thermal annealing treatment of the polymer. Later, Lulinski *et al.* [66] obtained a low level of template bleeding from a dopamine-imprinted polymer applying a Soxhlet extraction followed by a microwave-assisted extraction. More recently, other studies were performed to optimize the template removal from molecularly imprinted polymers [64,67].

To obtain high purification levels is also important to optimize the selective removal of the template fraction which is non-specifically bound to the imprinted matrix. An interesting study in this context was recently published by Székely *et al.* [68]. The authors combined the advantages of molecular imprinting and organic solvent nanofiltration for removal of 1,3-diisopropylurea (IPU, a potential genotoxic impurity) from mometasone furoate (an active pharmaceutical ingredient). In particular, a novel 1,3-diisopropylurea imprinted polymer was employed to remove residual IPU after nanofiltration of a mixture of IPU and API. The recovery of the API was achieved using two elution steps after the use of the imprinted polymer. The solvent methyl isobutyl ketone allowed recovery of the non-specifically bound API. Dichloromethane and methanol were used as secondary elution solvents to wash out IPU and regenerate the imprinted polymer [68].

The binding energies between the template molecule and the polymer/copolymer non-covalent interactions were studied by molecular dynamics [69–72] and quantum mechanical calculations, as the case of the frame of density functional theory (DFT) [73–75]. Recently, Prasad and Rai [76,77] used the second order Moller Plesset theory (MP2)/DFT to screen best functional monomers for specified template molecules. Theoretical results were in agreement with experimental data. In a different paper [78], a computational study was applied to investigate intermolecular interactions in the pre-polymerization mixture during the synthesis of an imprinted polymer towards 3,4-methylenedioxy-methamphetamine. The results of this study indicated that the best functional monomer and polymerization solvent for the preparation of the imprinted polymer were methacrylic acid and chloroform, respectively. Theoretical studies were also performed to investigate the selectivity of a cobalt imprinted polymer calculating the formation energies of the complexes between similar transition metal ions (Fe^2+^, Co^2+^, Cu^2+^ and Ni^2+^) and vinylbenzyl iminodiacetate as functional ligand [79].

Combinatorial chemistry was also adopted in order to accelerate the optimization of MIPs to attain the desired performance [80–82]. In combinatorial approaches the elements of the imprinting process (particularly the kind and molar ratio of the functional monomers) are varied using automated procedures [17]. In 2002, Chianella *et al.* [83] reported the synthesis of a computationally designed imprinted polymer for microcystin-LR. Later, a library of imprinted polymers was developed by Cederfur *et al.* that used the penicillin G as template molecule [84]. Assortment of the library was obtained by combining various functionalized monomers and cross-linkers and by changing the stoichiometry and the concentration of the components in the pre-polymerization mixtures. Recently, Khan and co-workers used a combinatorial screening procedure aiming the selection of polymer precursors in the preparation of the benzo[α]pyrene imprinted polymer [85].

**Figure 1.**
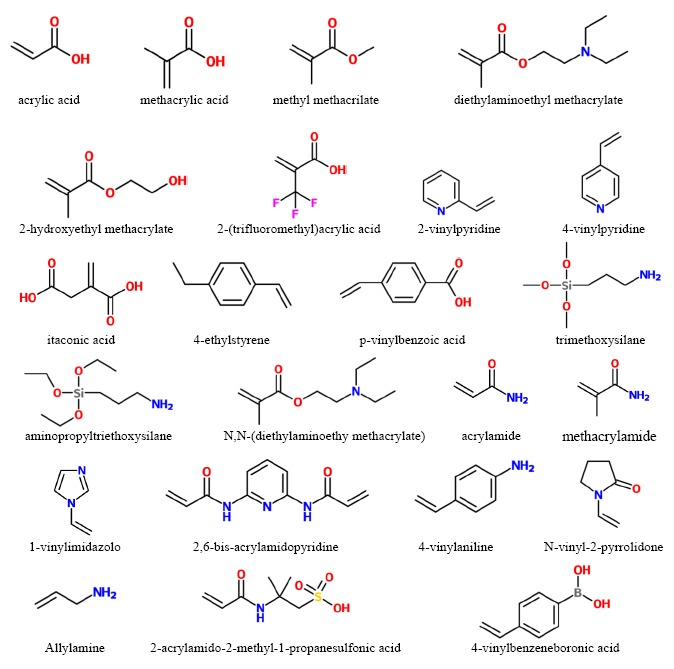
Some functional monomers used in MIP synthesis.

A detailed description of the different polymerization methods is beyond the scope of this review. However, it is important to highlight that free radical polymerization is usually the preferred method for preparing molecularly imprinted polymers. Methacrylic acid (MAA) and 4-vinylpyridine (4-VPY) are the most widely used functional monomers. The acrylic monomer MAA interacts via ionic interactions with amines and via hydrogen bonds with amides, carbamates and carboxyl groups. Owing to their strong force, the ionic interactions allow to obtain better performance of the MIP materials. The vinyl monomer 4-VPY is able to interact with the carboxyl functionality of template molecules. Other used functional monomers are 2-vinylpyridine, itaconic acid, acrylic acid, methyl methacrylate, 4-ethylstyrene, 1-vinylimidazole, *etc.* Two or more functional monomers may be also used together to improve the interactions with the template and, therefore the selectivity in recognition process. Figure 1 shows the chemical structure of some functional monomers.

**Figure 2.**
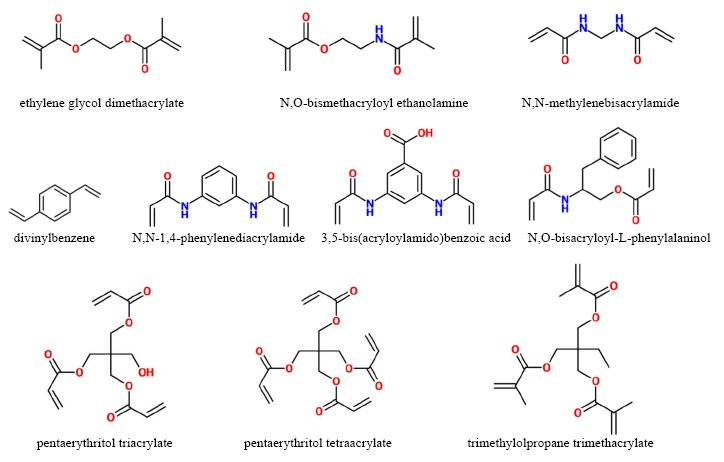
Some cross-linkers used in MIP synthesis.

The most used cross-linker is ethylene glycol dimethacrylate (EDMA) which contains two vinyl groups. Other cross-linkers, containing three or four vinyl groups were also exploited. It was found that they permit to obtain polymer exhibiting better load capacity and selectivity than the polymer prepared using EDMA [6,17,61]. Figure 2 shows the chemical structure of some cross-linkers used in MIP synthesis.

The morphology of imprinted polymers is very sensitive also to small alterations in the polymerization conditions. A useful method which permits control of the polymer's morphology is the “hierarchical imprinting approach” [86,87]. The target analyte is immobilized on the surface of a porous solid which acts as molds to create a desired porosity. Polymerization in the pores of the support and its subsequent removal allows to the creation of a porous solid containing surface-confined recognition sites specific for the template. This approach was used to imprint small molecules [86,88] and peptides [89,90].

Imprinted polymers are today well recognized in many areas which exploit their molecular selectivity. As an example, MIPs were applied in chromatography and chiral separation [91–93], in solid phase extraction [94–101], in enzyme catalysis [102], as antibodies and receptors mimics [103–105], drug delivery [106,107] and for macromolecules (e.g., proteins) recognition [108–111].

Even though the development of imprinted polymers has assumed great importance in all these fields, they have restricted processability due to the high cross-linking level (necessary to preserve the specificity) which renders them very hard and fragile materials. Anyway, owing to the integration of imprinting and membrane technologies, flexibility and specificity are achieved in a development of a special MIP format *i.e.*, “Molecularly Imprinted Membranes” which are stable and robust materials.

## 3. Molecularly Imprinted Membranes

A membrane is defined as a selective barrier interposed between two neighboring phases and regulates the transport chemical species amongst the two phases. The development of membrane technology dates back to many years before the coming of the molecular imprinting technique and was applied in many research sectors. For instance, membranes were used in water treatment [112], enzymatic catalysis [113], controlled drug release [114], oil refinement [115] and gas separations [116], for the development of biosensors [117] and so much more [118–120].

Taking advantage of the “bio-inspired” molecular imprinting technique, numerous research efforts were devoted to the production of a new generation of highly selective membranes named “Molecularly Imprinted Membranes”. Owing to the introduction of specific molecular recognition sites in its matrix, an imprinted membrane is able to discern between template and other analytes. These novel systems made valuable contributions in the development of innovative bio-mimetic molecular recognition devices. In fact, MIMs marked a new path for the detection, transport or retention of targeted chemical and biological compounds. Membrane-based imprinting processes do not require additives and can be performed at low temperature, thus reducing the energy consumption costs. In addition, compared to the traditional applications of imprinted polymers, MIMs can operate in a continuous mode by exploiting the characteristics of membrane and molecular imprinting technologies [11]. In comparison with a traditional membrane, a MIM exhibits an improved specific selectivity maintaining at the same time the separation efficiency.

Two diverse transport mechanisms of target molecules can be distinguished in a molecularly imprinted membrane [121]: the “*retarded permeation*” and the “*facilitated permeation*”. In the first case, the transport of the template through the membrane is retarded owing to the binding affinity with the imprinted sites distributed on the surface and bulk structure of the membrane. These kind of MIMs are generally macroporous and used as “*adsorber systems*” towards the analyte of interest allowing its separation from a mixture or microenvironment also containing other compounds such as contaminants, structural homologues, *etc.* The separation efficiency is strictly related with the binding capacity exhibited by the recognition sites. One example of imprinting of these systems is the development of imprinted nylon membranes for the selective adsorption of L-phenylalanine from L/D mixtures of this amino acid [122]. Yet, Malaisamy and Ulbricht prepared imprinted membranes for the selective adsorption of the biomarker Rhodamine B [12].

In the second type of transport (typical of micro-porous membranes), the passage of the template through the membrane is quicker and its perm-selective separation is achieved. This is due to the presence of a preferential path for the target molecules, which is produced via binding to and dissociating from neighbored recognition sites in the membrane facilitating their permeation. On the opposite, other solutes are subjected to the slow non-specific transport. An example of this transport mechanism was given from Chen *et al.* that developed an imprinted membrane exhibiting perm-selective properties toward the protein lysozyme with respect to bovine hemoglobin and cytocrom c [123]. Another example reports the preparation of luteolin-imprinted membranes, which exhibited very high selective transport of the template with respect to the similar rutin [124]. The selectivity factor α was 14.12.

This ability of MIMs to produce a selective transport or retention of specific molecules makes them good candidates for the development of highly innovative membrane processes. A wide variety of MIMs was successfully prepared exploiting the main following approaches: (1) the contemporary production of recognition sites and membrane structure of a self-supported membrane; (2) the synthesis of a tailored imprinted polymer to use in successive membrane preparation step; (3) the preparation of a composite imprinted membrane (by surface imprinting, thermal/photo-copolymerization of a pre-existing membrane).

The contemporary formation of membrane structure and molecular recognition sites is mainly accomplished by means of the “*in situ cross-linking polymerization*” and the so-called “*alternative molecular imprinting*”. The first method allows the formation of a cross-linked polymeric network produced by thermal or UV initiated polymerization of a mixture solution of template, functional monomer, cross-linker and initiator in a suitable solvent. Plasticizer agents are also added to the pre-polymerization mixture to obtain membranes that are more flexible. In 1990, Piletsky *et al.* [125] prepared the first flat-sheet imprinted membrane via *in situ* bulk cross-linking polymerization of acrylate monomers. In diffusion experiments, the membrane exhibited selective transport of adenosine monophosphate, a nucleotide that was chosen as the template.

Some years later Krotz and Shea prepared similar membranes by thermally initiated *in situ* cross-linking copolymerization using other nucleotides and nucleosides [126]. These membranes were freestanding, but fragile. Other authors attained an important improvement in terms of fragility and mechanical stability of freestanding membranes by adding to the “*in situ* imprinting” polymerization mixture an oligo-urethane-acrylate macro-monomer [127]. Enhanced permeability properties were also obtained simply by adding to the system a macromolecular pore former [128]. Imprinted membranes possessing enantio-selective recognition properties towards different amino acids [117,129] and pesticides [130] were also produced by using the same *in situ* polymerization method.

**Scheme 2.**
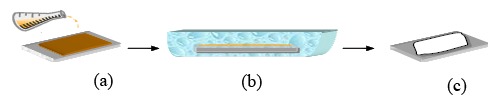
Schematic representation of a flat membrane formation process via phase inversion technique: (**a**) casting of the polymer solution on a support; (**b**) immersion precipitation; (**c**) formed membrane.

The alternative molecular imprinting is an expansion of a bio-imprinting in which existing recognition sites in an enzyme may be modified through the presence of a target molecule. According to this approach, candidate materials not having molecular recognition sites are transformed in molecular recognition membranes. The molecular memory of the template is induced in the membrane at the same time as the membrane is forming from its polymer solution. The process is mostly used for the preparation of membranes having flat-sheet configuration. The membrane is prepared in the presence of target molecules via the phase inversion technique. Briefly, a specific synthesized polymer (without cross-linker), which contains functional groups complementary to the template is used as membrane forming material. The process consists in the transformation of the polymer from the liquid state to a solid phase either by the “*dry*” or by the “*wet*” method. In the first method, the polymer solution is cast on a glass or Teflon plate and the polymer solidification is accomplished by the simple evaporation of the solvent. In the “*wet*” method, the film cast solution is immersed in a non-solvent and polymer solidification is attained by a precipitation induced through contact with the non-solvent [119,131]. Sometimes, a combination of the two methods is also carried out. Scheme 2 shows the preparation of a flat-sheet membrane via the “*wet*” phase inversion.

**Figure 3.**
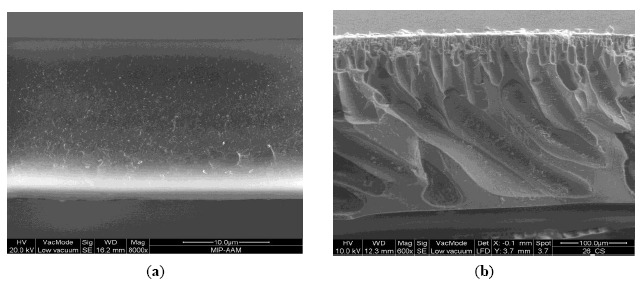
Cross-section of poly(co-acrylonitryle-co-acrylic acid membrane prepared via dry (**a**) and wet (**b**) phase inversion.

Normally, the “*dry*” phase inversion method allows obtaining membranes exhibiting a dense structure due to the progressive increase of the polymer concentration in the forming membrane owing to the solvent evaporation. Conversely, the “*wet*” phase inversion method leads to the formation of a porous membrane structure due to the quick liquid-liquid de-mixing typical of the non-solvent induced phase separation. Membranes can be prepared in a flat-sheet or hollow fibre configuration. Figure 3 shows the cross section of flat membranes prepared using the two different methods.

The “*dry*” phase inversion, was pioneered by Yoshikawa *et al.* that reported the preparation of molecularly imprinted membranes bearing oligopeptide derivatives, derivatives of natural polymers and other synthetic polymers as molecular recognition elements. They were applied in enantiomeric separation of amino acids and selective transport of nucleic acid components [132–141].

The “*we*t” phase inversion method was developed by Kobayashi *et al.* who prepared MIMs towards molecules of pharmaceutical interest such as the anti-asthmatic agent theophylline from blends of acrylate copolymers. The authors studied the influence of the coagulation temperature on the efficiency of the formation of the template-membrane's functional groups during the phase inversion process and on membrane selectivity [142–145].

Many researchers have successfully developed MIMs using the phase inversion imprinting method introduced by Kobayashi, which is now one of the most employed in this field. As well as acrylic copolymers [146,147] different polymers were used as membrane forming material, such as polyamide, polystyrene and polysulphone [148–150], cellulose acetate/sulphonated polysulphone blends [12], polyviniylidene fluoride and polyethersuphone [151], chitosan [152].

Most common prepared MIMs have flat-sheet configurations and are used for recognition of a wide range of compounds like pesticides [153], flavonoids [48,146,154], biomolecules like uric acid [13], vitamins [155] optical isomers [156–159] drugs [160] proteins [161] and much other. For example, Trotta *et al.* developed MIMs for the recognition of the antibiotic tetracycline hydrochloride. Membranes were able to selectively bound the template with respect of the structural analogue choramphenicol [162]. Fan *et al.* produced an imprinted membrane specific for the chemotherapeutic agent trimetophrim by blending polysulfone with a polymer imprinted with the target molecule [163]. Wang *et al.* prepared a metal ion imprinted membrane for the recognition and recovery of silver from waste solutions [164]. UI-Haq used the wet-phase inversion to prepared D-phenylalanine (D-Phe)- and L-phenylalanine (L-Phe)-imprinted membranes, which had a nanoporous structure. Applying these membranes for chiral resolution of phenylalanine a selective solute rejection was observed for the first time [165]. Wang *et al.* [166] developed MIMs with efficient perm-selective binding towards the template uracil.

The phase inversion process was also employed to introduce both size exclusion properties and molecular recognition sites during membrane forming step of molecularly imprinted nanofiltration membranes [10]. During nanofiltration in organic solvent, these membranes exhibited good recognition capacity and rejection performance.

Due to its feasibility the phase inversion technique was also exploited for the development of membranes by means of the so-called “*hybrid molecular imprinting*”. This strategy entails the dispersion of a pre-synthesized cross-linked imprinted polymer (*i.e.*, powders or micro/nano particles) into a normally used polymer matrix and the subsequent membrane formation via phase inversion. By using this method, Borrelli *et al.* incorporated imprinted polymer particles in a polymeric matrix. The obtained hybrid membrane was used for the selective depletion of riboflavin from beer [161]. Recently, Donato *et al.* developed hybrid imprinted membranes for the genotoxin 4,4′-methylene-dianiline [167]. Membranes were prepared by dispersing a pre-synthesized poly(acylonitrile-co-acrylic acid) imprinted polymer into polyvinylidene fluoride matrix. EDMA was used as cross-linker during polymer synthesis. The authors demonstrated an increase of the membrane performance with respect to previously developed non-hybrid membranes prepared directly with the imprinted polymer [168]. These membranes exhibited recognition properties in studies performed in organic environment. Wu *et al.* developed hybrid membranes for the separation of phenylalanine isomers incorporating an imprinted inorganic powder into a sodium alginate matrix [169]. To enhance the mechanical stability of sodium alginate and the compatibility between the two phases 3-aminopropyltriethoxysilane was used as precursor and cross-linking agent. Other publications deal with the preparation of hybrid imprinted membranes for the recognition in aqueous medium [170–180].

Imprinted microsphere and/or nano-particles may be also used for the preparation of composite MIMs by covering the surface of a pre-existing membrane *via* simple deposition or by means cross-flow filtration. Lehmann *et al.* [181] used imprinted microspheres as a filter cake between two polyamide microfiltration membranes, thus forming an enantio-selective multilayered composite membrane. Another example of membranes loaded with imprinted particles was reported by Silvestri *et al.* [182] that developed poly(acrylic acid-co-methylmethacrylate) membranes superficially modified by deposition of poly(methylmethacrylate-co-methacrylic acid)-based nanospheres imprinted with theophylline and caffeine. In a simultaneous application of the electrospray deposition and the phase inversion techniques, molecularly imprinted nanofiber membranes were also prepared for application in enantiomeric separation [183,184], selective separation of copper ions [185] and the alkaloid (−)-cinchonidine [186].

**Scheme 3.**
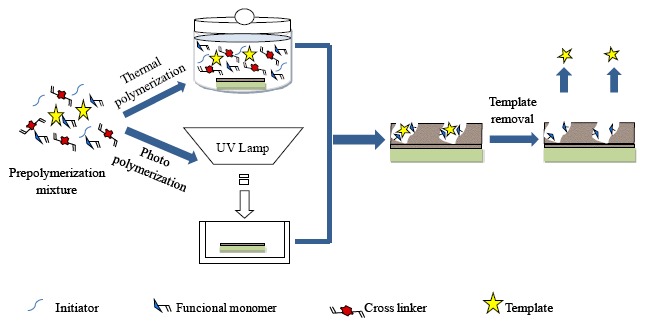
Illustration of the surface grafting via photo and thermal polymerization.

Composite MIMs were also prepared by other surface imprinting strategies, which entail the functionalization of the surface of a support membrane with a thin layer of molecularly imprinted polymer. In the presence of a template molecule, binding sites are introduced into the membrane support without damaging its pore structure. This method has the advantage to combine the mechanical integrity of the membrane support with the selectivity of the imprinted polymer. Two different polymerization routes may be followed: the surface grafting via photo-polymerization and the surface coating via thermal polymerization. The Scheme 3 shows the two different polymerization methods.

The membrane preparation process is accomplished by the immersion of a membrane to be modified in the pre-polymerization mixture and the subsequent polymerization. Depending of the polymerization method, photo or redox initiators are used to activate membrane surface by means of the formation of radical sites. Many flat sheet and hollow fibres membranes were developed by applying this method. First imprinted composite membrane was prepared by Wang *et al.* in 1997 [187] that used as support matrix a poly(acrylonitrile) membrane containing a photosensitive dithiocarbamate group. UV initiated graft copolymerization of acrylic acid and N,N methylene bis-acrylamide in the presence of theophylline as template allowed the formation of a molecularly imprinted layer on the membrane surface. Since then, many researchers prepared composite MIMs exhibiting high recognition performance. As example, Piletsky *et al.* [188] photographed flat-sheet polypropylene microfiltration membranes which showed recognition properties towards the herbicide desmetryn in water. Other membranes exhibiting selective permeation towards the template 4-amino-pyridine were prepared using the same method [189]. Recently, composite MIMs were produced by Liu *et al.* [190] for the selective recognition of the herbicide nicosulfuron for food safety detection. Koster and co-workers [191] coated silica fibers with a thin layer of methacrylate polymer for solid-phase microextraction. Donato *et al.* [52] prepared flat-sheet composite MIM by photo-copolymerization of polypropylene support with the functional monomer 4-vinylpiridine. This membrane is the first example of composite MIM which exhibited enantioselective permeation properties toward the anti-inflammatory drug *S*-naproxen. Composite membranes imprinted with *S*-naproxen were more recently developed *via* thermal copolymerization of the same functional monomer and the surface of hollow fibre polyvinylidene fluoride [192]. The separation properties of these novel imprinted membranes were determined by on-line HPLC membrane technology. Also in this case a selective permeation of the target molecules was observed. *S*-Propranolol-imprinted composite cellulose membranes were also prepared and employed as enantioselective-controlled release systems [193]. Polyvinylidene fluoride hollow fiber ultrafiltration membranes were also used as support for the deposition of a thin imprinted layer of poly(methacrylic acid). Applying the “template analogue strategy” composite MIMs were developed for the recognition of lovastatin acid in aqueous medium [194]. The process consists in the use of analogues complementary substructures of the template as “dummy template” exploiting the cross-reactivity of imprinted materials. This strategy avoids the problem of template leakage during its extraction in the imprinting process and/or template leakage in solid phase extraction [195]. It is also possible use this approach when target analytes cannot be directly used as template due to their toxicity or high costs and low availability. Zhai *et al.* [196] used the thermal polymerization method to prepare metal ion-composite imprinted membranes using as support material polyvinylidene fluoride. More recently, Zhu and co-workers used the same polymerization method to prepare composite MIMs able to selectively recognize magnolol, which is one of the most popular traditional Chinese medicines [197].

MIMs have been also produced by polymerizing thin imprinted polymer films on inorganic microporous glass-fiber membranes and Teflon filters [198] or on multiwalled carbon nanotubes [199]. The development of imprinted ultrathin titania gel films via surface sol–gel process was well reviewed by Kunitake and Lee in 2004 [200].

Some problems related to the preparation of MIMs are the limited accessibility of recognition sites owing to random distribution in the bulk and on the surface of the polymeric material. In addition, it is difficult to combine high yield imprinted sites with pore structure appropriate for efficient membrane permeability and separation.

In this section only some selected examples concerning the production of molecularly imprinted membranes are reported. Nevertheless, they symbolize the wide range of their application. In the next sections more attention will be given to the development of MIMs as molecular recognition elements for application in biosensors technology. For other information on molecular imprinting technology and applications, the reader is directed to some relevant publications [7,103,121,131,147,201–203].

## 4. Biosensors

**Scheme 4.**
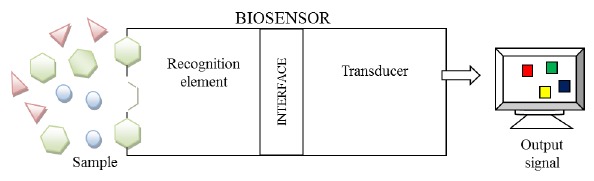
Scheme of a biosensor.

In the last two decades, biosensors have become very important tools for the detection of chemical and biological compounds for clinical [204,205] environmental [206,207] and food [208] monitoring. The wide range of applications in different fields of these devices is due to their excellent high specificity, sensitivity and rapid response. Biosensors comprise a biological recognition element and a transducer to convert the biological response into a measurable signal proportional to the analyte concentration [209,210]. In Scheme 4, a representation of a biosensor is shown. The biological recognition elements are classified in biocatatalytic and affinity [211]. The former type, (including enzymes, microbes, plant and animal cells and plant or animal tissue) provides a binding between the analyte and the biological species resulting in a change detected by the transducer. The latter type (including antibodies, receptors, nucleic acids and molecularly imprinted polymers) offers selective interactions of the sample with a specific ligand to form a thermodynamically stable complex [211,212].

The development of the biosensors depends on the immobilization methods of the biological macromolecules for solving several problems such as their loss, preservation of their stability and shelf life [213,214]. To reach these objectives, different immobilization techniques were investigated such as physical entrapment, adsorption, covalent binding and covalent cross-linking [215]. It is necessary to mention that the selected immobilization method depends on the nature of the biological element, type of the transducer and operating conditions of the biosensors [216]. Physical entrapment is the oldest and a very simple method, but with different drawbacks as the loss of the biological element as the pH varies [217]. The most used methods are those that involve the formation of a chemical bond. In particular, the bond is formed between the functional group of the biological species and the reactive group of the support. The biosensors can be also classified in different types in relation to the transducers: electrochemical (potentiometric, amperometric, impedimentric, conductimetric), calorimetric, piezoelectric and optical transducers. Electrochemical approaches are extensively used in the development of these devices. Potentiometric transducers measure the potential difference between a working electrode and a reference electrode, and the signal is correlated to the concentration of analyte [218]. The sensitivity and selectivity of these biosensors are excellent. However, a reference electrode very stable and accurate is required and consequently it could represent a limitation. The amperometric biosensor-type measures the current produced by the reduction or oxidation of an electroactive species present on the surface of a working electrode [219]. They are accurate and precise. Nevertheless, they are less sensitive because the measured current is only governed by the redox potential of the electroactive species so the contributions of other chemical species can be included in the measurement result [220]. Conductimetric sensors work based on the change of conductivity associated with many reactions that imply a change in ionic species [221]. They are less used because are not specific and at the same time present a very low signal/noise ratio [220]. Recent studies on biosensors have demonstrated that electrochemical impedance spectroscopy (EIS) detection is much preferred to other electrochemical methods including amperometric and potentiometric ones [222,223]. EIS is a characterization technique that offers electrical information in the frequency domain [224,225]. With this technique, a process takes place in an electrochemical cell and it can be modelled using a combination of resistors and capacitors [226]. However, a major disadvantage of EIS methods is their low sensitive detection limit compared to the other technologies for the lack of a signal amplification method adaptable to the EIS [227]. Biosensors with thermal transducers exploit the change of heat that occurs during chemical reactions [228]. Anyway, the heat is not confined in an adiabatic system and this determines a loss of information due to any partially loss of the heat by irradiation, conduction or convection [228]. Piezoelectric biosensors have a biological recognition agent immobilized on a surface of a piezoelectric element (for example a quartz crystal) [229]. The presence of the analyte can be detected by immersing the piezoelectric crystal in the reaction solution to allow a bond between the target species and the immobilized biological element [230]. This determines a modification of the crystal mass with a consequent change in the vibrational frequency [231]. The drawbacks of a piezoelectric sensor are the lack of an exact correlation between mass addition and frequency change for solution phase and sensitivity to environmental conditions. Is important to note as optical biosensors are receiving much attention due to the developments in the optical fiber technology. The variation of the optical properties such as UV-Vis absorption, bio-and chemo-luminescence, reflectance and fluorescence is caused by the interaction of the analyte with the biological element [231]. They are compact, flexible and present a small probe size.

Following are reported some recent and interesting examples of biosensors enzyme-based for the detection of pesticides. Acetylcholinesterase (AChE) is an enzyme that stabilizes the levels of the neurotransmitter acetylcholine for the catalytic hydrolysis of acetylcholine to thiocholine [232]. The AChE catalytic activity is inhibited by trace amounts of organophosphorus pesticides (OPs). Biosensors based on AChE are applied for the detection of pollutants such as OPs and carbamates. Recently, Guan *et al.* [233] developed a novel (AChE) biosensor based on multilayer films containing chitosan and AChE liposome bioreactor. The biosensor was not expensive; it showed high sensitivity, and good reproducibility resulting capable to detect pesticides.

**Figure 4.**
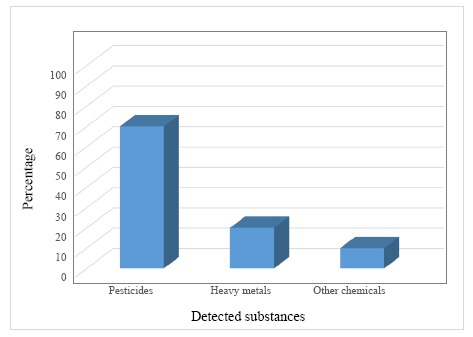
Applications of enzymatic biosensors. (Data from [214]).

A very large number of enzyme biosensors is studied for the detection of pesticides. In particular, as it is shown in Figure 4, about 70% of these devices is employed for the determination of pesticides, the 20% is applied in the determination of heavy metals and the rest (10%) for other chemicals (benzoic acid, nitric oxide, cyanide and so on) [214].

Creatinine is an important clinical analyte for the diagnosis of renal and muscular dysfunction [234]. Creatinine is detected by using both the colorimetric method by means of the Jaffe reaction [235] and enzymatic colorimetric methods [236]. However, the two different methods present different drawbacks [237]. The biosensing method presents different advantages with respect to the traditional techniques in terms of time reduction, simplicity, sensitivity and analysis costs. For example, Yadav and co-workers [238] demonstrated the possibility of using iron oxide nanoparticles/chitosan graft-polyaniline (Fe_3_O_4_-NPs/CHIT-g-PANI) composite film for construction of an amperometric creatinine biosensor. The biosensor showed rapid response, higher sensitivity, good reproducibility and long-term stability. The very expensive and time-consuming enzyme purification process hinders the application of the biosensors-enzyme based at large scale. Microbes (algae, bacteria, and yeast and so on) offer an alternative in the manufacturing of biosensors because they can be massively produced by cell culturing. They present other advantages as the ability to identify a large variety of analytes and the possibility to work at wide range of temperature and pH [239]. Singh and Mittal [208] demonstrated as a biosensor obtained by immobilizing *Chlorella sp.* microbes over a glassy carbon electrode are highly selective for mercury without interference from alkali, alkaline earth and other transition metal ions. The experimental results showed high reproducibility with a lifetime of 14 days. An amperometric biosensor based on the immobilization of a microbe (*Pseudomonas alcaligenes* MTCC 5264) on a cellophane membrane detected caffeine in solution over a concentration range of 0.1 to 1 mg·mL^−1^ in a very short time (3 min) at pH of 6.8 and temperature of 30 °C [240]. However, in this study sugars like glucose and sucrose gave interference in analysis of caffeine. An atrazine imprinted polymer was prepared by Lavignac *et al.* using a non-covalent approach [98]. More recently, Yaqub *et al.* [241] developed artificial receptors via *in situ* MIP synthesis directly on gold electrodes of piezoelectric transducers using as template the same pesticide.

The principal drawbacks present in the biosensors field are the chemical and physical properties of the biological recognition elements being unstable when are not in their natural environment and this results in difficulties to fine-tune them for a specific application [242]. In this context, molecular imprinted polymers, which were meticulously discussed in Section 2, can represent valid substitutes for the biological agents owing to their peculiar characteristics [243]. MIPs are combined with different type of transducers (optical electrochemical, acoustic, *etc.*). Recently, a molecularly imprinted polymer was developed and integrated in an optical sensor for the detection of the antibiotic enrofloxacin [244]. The authors demonstrated the capability of the MIP to selectively recognize the target molecule. Gonzalo *et al.* [245] prepared an opto-sensor based on an imprinted polymer synthesized using toluene as template. The sensor was used for the screening of a mixture of toluene, ethylbenzene and xylene in drinking water. During the test the contaminated samples were identified quickly (81 s) with a cut-off level of 700 μg·L^−1^ for ethylbenzene, which is the maximum level established by US Environmental Protection Agency [245]. In recent times, the possibility to determine 2,4-dinitrophenol in water samples by using an electrochemical sensor based on an hydrophilic imprinted polymer was demonstrated [246]. The novelty of the work is represented by the presence of a hydrophilic imprinted polymer that could enhance the accessibility of the target species to the imprinted cavities and thus improve the selectivity of the MIPs in a water medium.

## 5. MIMs-Based Bio-Mimetic Sensors

Biosensor technology is an area characterized by novel testing approaches. Since the first biosensor was developed by Updike and Hicks in 1967 [247] many biosensors have been studied and developed. Classical molecular biorecognition materials are based on the use of biological molecules such as antibodies, enzymes, microorganisms, as recognition elements. Despite their high selectivity, biosensors represent today only a small proportion of the market due to problems directly related with the chemical and physical properties of the biomolecules employed. In fact, these biological receptors possess high selectivity but owing to their poor stability, it is difficult to incorporate them into devices for developing biosensors. In addition, they suffer of availability, environmental intolerance and reproducibility. In the last decades, biosensing systems using molecularly imprinted materials as recognition elements instead of biomolecules have received attention of scientists in various fields due to their potential facility to overcome some of these problems. Traditional applications of imprinted materials in sensor technology involved the use of imprinted polymer particles. Nevertheless, the interface adhesion between the particles and the transducer surface can be poor. In addition, the response time is extremely long, owing to the slow mass transfer [9], so as an alternative to imprinted polymers, a special format of them like thin films or molecularly imprinted membranes are attracting growing interest.

In fact, the direct and rapid determination of an interaction between the recognition agent and the target analyte represents an encouraging factor for the development of MIMs as alternatives to the traditional bioassay methods. This is because they don't need the addition of secondary reagents and the separation of free and bound reactants [15]. In addition, MIMs-based sensors take advantage of their high selectivity and stability, which enable them to long-term operation under conditions not tolerated by biomolecules without losing sensitiveness. They can operate in acid/basic solutions, high temperature, organic environment, *etc.* Furthermore, MIMs can work in a continuous way and are therefore promising for an industrial application.

Hedborg *et al.* [248] that developed an L-phenylalanine-imprinted polymeric membrane in combination with a field-effect capacitance device performed first studies on this topic. The authors demonstrate that the change in capacitance of the system was similar for the template and its structural analogue tyrosinanilide, while the similar phenylalaninol determined only a small change of capacitance in the system. The recognized template was detected by means of selective binding. Alternatively, Piletsky *et al.* developed a sensor device based on the selective permeation of an analyte through an imprinted membrane, which was able to recognize low-weight organic molecules [249].

In some MIMs-based sensors, also developed by Piletsky *et al.*, an opposite response between covalent and non-covalent bonding-based sensors was observed. In particular, an increase of template concentration caused a reduction in conductivity signal in covalently-imprinted device, while opposite behavior was observed for the non-covalent one. This different response was attributed to the presence of more numerous and homogeneous recognition sites in the covalent systems, which shrank more reducing the micropores when the template was added. Probably this phenomenon determined a decrease of the electroconductivity based on ion transfer [250].

Up to now, different sensor-based imprinted membranes were developed. The most common fields of their application are the detection of contaminants in water and food, the analytical sensing of bioactive molecules as well as metals detection. In the following sections, some relevant examples are discussed.

### 5.1. Detection of Water Contaminants

The development of MIMs-based sensors for the detection of toxic (and also warfare) chemical agents has a strong impact in the protection of human health. Very important is the possibility to also detect low levels of water contaminants and enable the implementation of protective interventions.

Piletsky *et al.* developed a MIM-based-conductometric sensor for the detection of atrazine in water. Membranes were supported on a glass filter. This sensor was able to recognize triazine in the range of 0.01–0.5 mg·L^−1^ and was reused for several months without losing its sensitivity [251].

Atrazine-imprinted membranes, containing artificial recognition sites for atrazine were also prepared by photopolymerization of the template, using methacrylic acid as functional monomer and tri(ethyleneglycol) dimethacrylate as cross-linker. To obtain thin, flexible and mechanically stable membranes, oligourethane acrylate was added to the monomer mixture [127,252,253]. Pogorelova *et al.* developed imprinted acrylamide-methacrylate co-polymer films by electropolymerization of Au-quartz crystals for the selective detection of atrazine and other triazines like prozinex, ametrex, terbutex, simanex, tyllanex [254]. The specificity of the recognition sites was attributed to the complementary electrostatic interactions and hydrogen bond between the target analyte and the imprinted film. More recently, D'Agostino *et al.* developed new atrazine-imprinted membranes integrated with a potentiometric sensor [255]. The membrane was directly created at the end of a small Teflon tube which was used for assembling the sensor. The detection limit was around 2 × 10^−5^ mol·L^−1^. The technique of grafting polymerization was used for preparation of thin films of molecularly imprinted polymers on the surface of polypropylene membranes and on hydrophobized gold electrodes. The herbicide desmetryn was used as a template [256]. The same technique and template were used some years later for the development of an ultrathin chemo-sensor. An adsorbed layer of a hydrophobic photoinitiator (benzophenone) conferred graft polymerization on the surface of gold electrodes for the development of alkane-thiol-modified gold electrodes. Binding of the target molecules allowed a reduction of the dielectric constant of the polymeric layer. The amount of bound analyte was detected by measurements of electrical capacitance [257].

**Figure 5.**
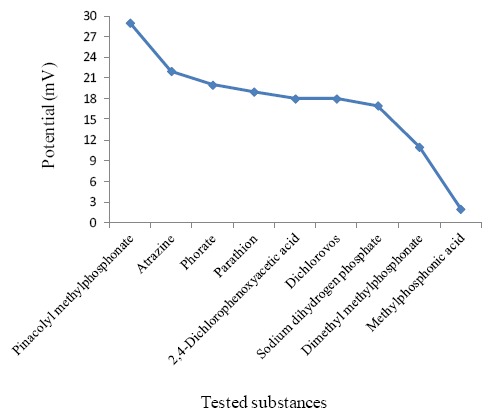
Potentiometric response of the PMP bulk-polymerization based-sensor toward pinacolyl methylphosphonate (PMP) and other common contaminants (Data from [260]).

Sergeyeva *et al.* [258], by mimicking for the first time the active sites of the enzyme tyrosinase in a freestanding MIM, constructed an easy-to-use biomimetic sensor for the detection of phenols in water. Concentration of phenols in the analyzed samples was detected using a universal portable device oxymeter with the oxygen electrode in a close contact with the catalytic imprinted membrane as a transducer. The detection limit was 6.3 × 10^−2^ mM. A biomimetic potentiometric field monitoring device was also developed for the detection of traces of the insecticide phorate (O,O-diethyl-S-ethylthiomethyl phophorodithioate) in natural waters. The sensing element was produced by the inclusion of a phorate-imprinted polymer in a polyvinyl chloride membrane. The detection limit was 1 × 10^−9^ M. The applicability of this new sensor for analyzing ground, river and tap-waters was demonstrated and it was promising for routine monitoring of phorate [259]. Furthermore, pinacolyl methylphosphonate (PMP), a degradation product and an active analogue of soman (a highly toxic nerve agent) was chosen as template to evaluate the sensing performance of potentiometric sensors which employed MIMs prepared by bulk, precipitation and suspension polymerization methods based on non-covalent molecular imprinting [260]. Different sets of membranes were prepared. The sensor response behaviour was based on the average of potential outputs obtained with each membrane. The imprinting effect was more predominant in the case of bulk polymer based-sensor which exhibited better sensitivity and selectivity. This result was attributed to a more rigid polymeric structure leading to more stabilized recognition sites. Figure 5 shows the potentiometric response of the PMP bulk-polymerization based-sensor tested for sensing PMP and some other common contaminants.

As it is evident, the higher potential response was observed in the case of PMP. Owing to the imprinting effect, the performance of this sensor (which was useful for routine monitoring of traces of PMP in ground and tap-water samples), in terms of sensitivity, selectivity and stability was superior to other sensors [260].

Apart the examples described above, many other MIMs-based biomimetic sensors were developed for the detection of pesticides [256,261–263], haloacetic acids [264,265] and other organic pollutants as discussed elsewhere [151,266,267].

As it is known antibiotics are “pseudopersistent” contaminants owing to their continuous entry into the ecosystem and high sensible detection devices are required for their early detection, thus preventing toxic effects. Chianella *et al.* [268], reported a combination of molecularly imprinted solid phase extraction (SPE) cartridges (as sensing elements) with a MIP-based piezoelectric sensor for the determination of microcystin-LR, which is one of the most prevalent and dangerous toxins produced by freshwater cyanobacteria. This was the first example where the same imprinted receptor was used for both solid-phase extraction and the matching sensor. The minimum detectable amount of toxin was 0.35 nM. Rebelo *et al.* proposed a new biomimetic sensor based on imprinted membranes for the detection of the antibiotic trimethoprim, a synthetic antibiotic used in human and veterinary medicine [269]. Membranes were prepared by dispersing in a polyvinylchloride matrix and imprinted polymer synthesized using alternatively methacrylic acid and 2-vinylpyridine as functional monomer. The process was accomplished with the aid of *o*-nitrophenyl octyl ether as a plasticizer. Recognition experiments indicated that the imprinted material acted as ionophore in a charged carrier mechanism. In aquaculture waters, the membrane containing 1% of MIP/MAA imprinted polymer showed better analytical response in terms of accuracy and selectivity with respect to an electrode prepared with a traditional ion-exchanger [269]. A similar MIM-based membrane was also developed for enrofloxacin [270] and chlortetracycline [271].

An electrochemical sensor based on a MIM for 17β-estradiol was constructed on the surface of platinum nanoparticles-modified glassy carbon electrode (PtNPs/GCE). 17β-Estradiol is a steroid present in many environmental media and particularly in water. Low concentrations of this substance may cause the disequilibrium of the humoral and cellular immunity, resulting in many pathological situations. Capacity tests performed to determine 17β-estradiol in real-time water samples demonstrated an exceptional high immunity against matrix interference. The detection limit of 1.6 × 10^−8^ mol·L^−1^ [272]. More recently, a MIM-based biomimetic sensor was proposed for probing rivastigmine in tablets and biological fluids. This compound is an acetylcholinesterase inhibitor of the carbamate type agreed for the treatment of Alzheimer's disease. The sensor was successfully used for the determination of rivastigmine concentration in human serum, plasma, urine, rat brain and tablets. It exhibited good performance and discriminated other antibiotics [273]. Sener *et al.* [274] attached nanoparticles imprinted with lysozyme on the surface of surface plasmon resonance sensor. The nanosensor exhibited the ability to detect template molecules from both aqueous and natural complex source, chicken egg white at low concentration (32.2 nM).

Molecularly imprinted TiO_2_ thin films on ion-sensitive field-effect transistors were also used to prepare sensors for the detection of chloroaromatic acids [275], thiophenols and benzylphosphonic acids [276] and carboxylic acids [277].

### 5.2. Sensing of Drugs and Bioactive Molecules

In 1999 the Piletky group [278] developed novel molecularly imprinted thin films in an amperometric sensor. The films were prepared by spontaneous self-assembly of hexadecylmercaptan in the presence of cholesterol (as template) on a gold electrode. The sensor was prepared by co-immobilizing the template and the functional monomer by simple sorption on the electrode surface and subsequently washing the system to extract the template. The removal of cholesterol allowed the formation of recognition sites able to rebind it. During binding tests (performed in 50% aqueous ethanol), the molecular recognition that occurred reduced the mass-transport of the electroactive potassium ferrocyanide to the electrode surface and consequently the current changed. The change of the potassium ferricyanide reduction peak was related to the template concentration: the peak value decreased with increasing cholesterol concentration. As it is well known, the reduction of human blood levels of cholesterol and low density lipoproteins is very important to fight the atherosclerosis disease, so it is extremely relevant to have the possibility to control the blood cholesterol level. Recently, Schirhagl *et al.* developed an artificial receptor by surface imprinting of polyurethane with insulin crystals [279].

In a different way, Ciardelli *et al.* developed composite MIMs useful for the selective recognition and removal of cholesterol in an extracorporeal blood treatment. The employment of these membranes could be an alternative to the traditional methods which use antibody or other chemical adsorbents as recognition elements [280]. To prepare the composite membrane cholesterol-imprinted nanospheres made of poly(methacrylic acid) were simply deposited on the surface of a membrane made of a (methylmethacrylate-co-acrylic acid) copolymer prepared via “the phase inversion technique”.

**Figure 6.**
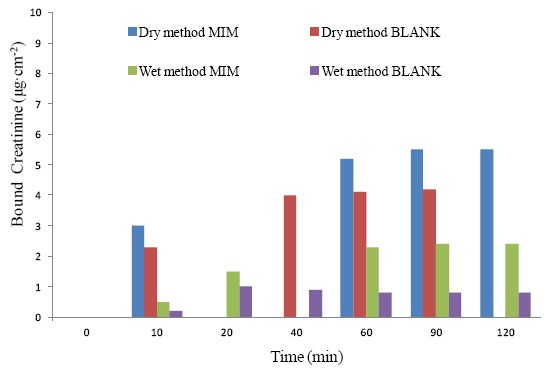
Rebinding of creatinine to MIM and BLANK membranes (Data from [281]).

This technique was also used to develop poly(ethylene-co-vinylalcohol) (EVAL) MIMs as bio-mimetic recognition element for the detection of creatinine and to determine kidney dysfunction [281]. Recognition experiments, performed in aqueous solutions, showed that after ten minutes half of all binding sites were occupied for MIM and BLANK membranes synthesized by either “wet” or “dry” phase inversion. The binding of creatinine reached the equilibrium in the first hour of incubation (see Figure 6).

Selectivity tests, performed in real urine samples in comparison with biomolecules also present in blood and urine (albumin, uric acid and creatine) showed that the binding of creatinine was 9.0-fold, 4.5 fold and 3.0 fold with respect to creatine, albumin and uric acid, respectively. The creatinine-imprinted membrane seemed to be promising for the integration with a quartz crystal microbalance for developing a portable homecare system useful for the non-invasive diagnosis of kidney function or aging of human body thus improving elderly life quality [281]. Lobo-Castañón and coworkers developed electrochemical sensors with electrodes modified with thin imprinted polymer films. Different modification strategies were investigated: electropolymerization, preparation of poyphosphazene-modified electrodes, immobilization of acrylic imprinted polymer particles on the electrode surface with polyvinylchloride, spin-coating of acrylic membranes [282–284]. MIMs may be also valid substitutes for natural receptors used in immunoassay methods [285]. Surugiu *et al.* [286] developed for the first time an imaging assay analogous to competitive enzyme immunoassays using a molecularly imprinted recognition agent instead of an antibody. Polymer microspheres imprinted with 2,4-dichlorophenoxyacetic acid were used as cover layers of microplates using polyvinyl alcohol as fixing agent. The template was labelled with the tobacco peroxidase enzyme. In a competitive method the bound fraction of the conjugate was quantified by monitoring the chemi-luminescence reaction of luminol. Light emission was measured in a high-throughput imaging format with a camera. This system can be very useful when it is difficult to obtain antibodies or it is necessary to reduce assay costs.

In a different approach [287] transparent films as recognition elements were produced by chemical grafting of the imprinted homopolymers of 3-aminophenylboronic acid to the surface of microplates made of polystyrene. Epinephrine, a natural ligand of the adrenergic receptor was used as template. Owing to its chemical structure, this molecule presents different functional groups, which are suitable to interact with the functional monomer depending on the pH: catechol group in the aromatic ring (covalent interaction), hydroxyl group, and secondary amino group.

The thin films were applied as artificial adrenergic receptors in Enzyme-Linked Assay (ELISA) for the determination of β-agonists using a conjugate of horseradish peroxidase and norepinephrine (HRP-N). The binding properties of the imprinted device were determined by monitoring the competitive adsorption between the free template and the HRP-N conjugate. It was found that the imprinting process allowed increasing the polymer affinity toward HRP-N and epinephrine [287]. This case represents an anticipation of the hopeful application of imprinted recognition elements in diagnostic assay and drug screening.

Electronic transducers were associated with acrylamide-phenylboronic acid-acrylamide copolymer membranes imprinted with nucleotides and monosaccharides [288]. The imprinted membranes were assembled on piezoelectric Au quartz crystals or Au electrodes via electropolymerization or on the gate surface of an ion-sensitive field-effect transistor device by radical polymerization. Microgravimetric quartz crystal microbalance measurements, Faradaic impedance spectroscopy, and ion-sensitive field-effect transistor devices were employed to assess and transduce the association of the each template (nucleotides adenosine5′-monophosphate, guanosine 5′-monophosphate, cytosine 5′-monophosphate and uridine 5′-monophosphate) with the corresponding imprinted membrane. The specific sensing of nucleotides of these devices is interesting for the development of new rapid sequencing methods for the nucleic acids DNA and RNA [288].

A new sensor for the detection of NAD(P)+/NAD(P)H cofactors with employed imprinted polymer membranes associated with ion-sensitive field-effect transistor devices and Au-quartz crystals was proposed by Pogorelova *et al.* [289]. The oxidized cofactors NAD^+^ and NADP^+^, and the reduced cofactors NADH and NADPH were used as templates. Authors demonstrated the selective sensing of each template and the application of the functional devices to follow biocatalyzed transformations such as oxidation of lactic acid and ethanol in the presence of lactate dehydrogenase and alcohol dehydrogenase [289].

Multiwell glass-fiber membrane filter plates modified with imprinted polymer were also developed for the detection and selective removal of the β-blocker drug propanolol from blood and urine samples. Filter plates were modified by photo-copolymerization with methacrylic acid as functional monomer and EDMA as cross-linker [290].

A potentiometric sensor for the selective detection of the biomarker of endospores dipicolinic acid (DPA) was developed by surface imprinting of a polysiloxane film which was integrated with a nanoscale transducer. The detection of DPA was in the concentration range of 1.5 × 10^−6^ M to 1.94 × 10^−1^ M [291]. The sensor response time for 4 × 10^−4^ M of DPA was 25 s. This sensor was promising as portable device for rapid and emergency determination of potential biological weapons.

A composite MIM was also prepared on a cellulose acetate support by the photopolymerization of methacrylic acid and a cross-linker, ethylene glycol dimethacrylate. The cytokinine 6-benzyladenine was used as template. Rebinding studies demonstrated as these membranes were able to selectively bound the target molecules, which may be applicable for assaying plant samples [292]. Composite MIMs have been also suggested by Pectu for sensing the anesthetics in blood. Propofol (2,6-diisopropylphenol) was chosen as a model template. Membranes were prepared by polymerizing a thin-imprinted polymer film on the surface of polytetrafluoroethylene, cellulose and nylon membranes. The total time assay of sensing tests carried out on real blood solutions was 3 min. The membranes exhibited good linearity and specificity at clinically significant concentrations of 1–10 μg·mL^−1^. These results indicate the suitability of these membranes as sensor elements in an on-line bio-mimetic sensor [293]. In 2006, Chen and coworkers used the new functional monomer 9-vinyladenine for the preparation of composite MIMs showing high permselectivity towards the template *H*-indole-3-acetic-acid [294], a plant hormone which regulates plant growth. The characteristics of these membranes seem to render them applicable for the detection of this organic acid in plant samples

Kobayashi *et al.* [295] were the first to develop a quartz-crystal microbalance (QCM) sensor employing an imprinted membrane for the detection of the stimulant drug caffeine. The authors used the phase inversion precipitation method to prepare two imprinted poly(acrylonitrile) copolymers using pyridine and styrene as functional monomers. The prepared membranes were applied as novel recognition elements covering the surface of an Au electrode. The recognition properties of this new sensor device were assessed by monitoring the frequency change of the system after template binding [295].

Yu *et al.* [296] develop a voltammetric sensor based on MIM-modified electrode by creating a membrane layer (via photopolymerization) on the surface of a glassy carbon electrode (GCE). Emodin, an active ingredient of traditional Chinese medicines was used as template. Allobarbital was used as a novel functional monomer which was able to establish multiple hydrogen bonds with the template. The novel sensor was tested for the determination of emodin content in Sanhuang tablets and compared with classical HPLC measurements. Results of these methods were about 2.49 × 10^−1^ (mg·tablet^−1^) and 2.46 × 10^−1^ (mg·tablet^−1^), respectively [296].

An electrochemical sensor based on chitosan MIM film was also build for the detection of urea which is an indicator of abnormal human physiological conditions. Sensing experiments showed high linear sensitivity to urea in the range from 1.0 × 10^−8^ to 4.0 × 10^−5^ M with a detection limit of 5.0 × 10^−9^ M and a recovery range from 96.3% to 103.3%. These characteristics emphasized the great potential applications of the device for early-stage clinical diagnosis in blood serum [297].

A sensing system for the rapid detection of ractopamine in pig urine was constructed by directly synthesizing MIMs (via an *in situ* thermal polymerization technique) on screen-printed electrodes modified with multi-wall carbon nanotubes (MWCNT) and connecting it with an electrochemical analyzer. Ractopamine is an artificial β-agonist which promotes animal growth. Sometimes it is illegally used in substitution of clenbuterol or salbutamol resulting in hazardous effects to human health, so for food safety its rapid detection in animal samples is very important. The novel sensor allowed the quantification of ractopamine in pig urine. The assay time was within 5 min and the detection limit was 6 nM [298]. A recent paper deals with the development of a MIM-based liquid and grafite selective electrodes in poly(vinyl chloride), matrix membranes for the quantification of the dextromethorphan in antitussive and cold syrups. These novel electrodes showed short response times, good stability, sensitivity and selectivity and lifetimes of more than three months. In addition, they had a stable potential in the pH range from 2.0 to 9.0 [299].

An emerging field in the area of molecular imprinting is the sol-gel technique which combines imprinting technology and the sol-gel process. The latter is a versatile method to entrap sensitive organic, organometallic and biological molecules in porous ceramic materials. It allows also the design of new materials with tailor-made pore sizes and shapes for specific analyte recognition.

In general, the sol–gel process involves the transition of a system from a liquid “sol” (mostly colloidal) into a solid “gel” phase. In particular, progressive polycondensation reactions of molecular precursors in a liquid medium allow the construction of an oxide network. The most common precursors are tetramethyl- and tetraethylorthosilicate [300,301]. Molecular imprinting of sol–gel thin films is advantageous over organic-polymers, because of its easy preparation, low non-specific adsorption and high association constants. Many research groups have investigated the possibility of integrating sol-gel films as sensing elements–with electrodes as transducers. As example, Fireman-Shoresh *et al.* demonstrated the chiral imprintability of spin coated submicron composite sol–gel films (∼700 nm) and their enantioselectivity under steady-state conditions for various enantiomers [302,303]. Sol-gel imprinted membranes were prepared by Almeida *et al.* [301] that imprinted the antibiotics sulfadiazine (SDZ) and sulfamethoxazole (SMX) in sol-gel materials which were used as sensing elements included in the matrix of poly(vinyl chloride)-based membranes connected to a potentiometric transducer. SDZ is used to treat toxoplasmosis and to put off some type of meningococcal meningitis. SMX is used for the treatment of bacterial infections. The use of these substances as antimicrobials in aquaculture environments presents serious hazards owing to food, aquaculture water and environmental contamination. The sensors were evaluated in steady-state conditions and applied to the analysis of drugs, water and biological samples. The analytes were recognized by means electrostatic interactions with the silica groups of the sol–gel membrane matrix. The detection limits were 0.74 μg·mL^−1^ and 1.3 μg·mL^−1^ for SDZ and SMX, respectively [301].

Sol-gel imprinted polymer films were also developed for the detection of parathion in liquid and gas phase using cyclic voltammetry (CV) and QCM, respectively [261]. L-Histidine was also imprinted on sol-gel thin films [304]. Finally, an interfacial organic-inorganic hybridization concept was applied to the preparation of a new spherical imprinted material for recognition of BSA [305]. The authors evaluated the influences of pH on the resulting template removal and readsorption characteristics. At pH 2, the material possessed low bovine serum albumin readsorption capacities. At pH 7, removal of BSA was highest, whereas readsorption was the lowest [305].

Molecularly imprinted materials prepared via sol–gel process have great potential for different sensing applications, particularly for environmental monitoring.

### 5.3. Detection of Food Additives and of Metal Ions

**Figure 7.**
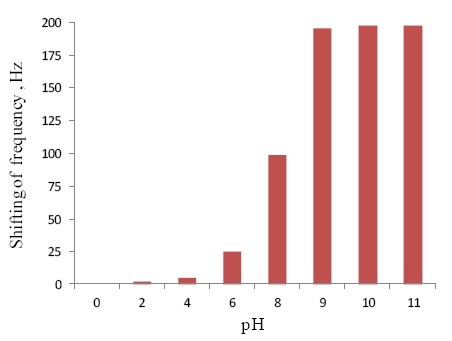
Influence of pH on the sensor response during determination of vanillin in food samples (Data from [306]).

Avila *et al.* [306] developed an on-line supported liquid membrane-piezoelectric detection device, based on a molecularly imprinted polymer manifold, as a new method for the quantification of vanillin, one of the most used flavours in foods, beverages and confectionery. The supported liquid membrane extraction technique is based on a three-phase system with an organic phase sandwiched between two aqueous phases. The organic phase is immobilized in a porous hydrophobic membrane. The analyte is extracted from a donor phase into the hydrophobic membrane, and then back extracted into a second aqueous phase used as the acceptor solution [306]. The separated vanillin was transferred in a piezoelectric flow cell which transmitted to a frequency counter and the finally recorded the signal. The desorption time after vanillin absorption did not exceed 10 min, which is an acceptable time for sensors. The use of a supported liquid membrane coupled on-line to a quartz crystal microbalance based on an imprinted polymer enhances selectivity by avoiding interference from matrix constituents. Owing to the hydrogen bonding, as well as electrostatic interactions and charge transfer in the template-functional monomer (methyl-methacrylate) bond, the pH had great influence on the sensor response. As shown in Figure 7, an increase of pH from 1.0 to 9 allowed also an increase of the frequency signal. At pH value ranging from 9 to 11 the frequency signal was almost constant, indicating that the system was stabilized [306].

In a previous paper, the same authors developed a similar sensor for the detection of caffeine in real coffee and tea samples [307]. Also in this case the authors used the advantages of the SLM technique such as analyte enrichment and proficient removal of interfering agents, thus improving the performance of the sensor with respect to an already developed device for caffeine which used only an imprinted polymer [308].

A nicotinic acid (NA) sensor was also produce by creating a thin imprinted membrane layer on the surface of a glassy carbon electrode. NA is widely used in medicine and food additives. The sensor exhibited high selectivity for the template with respect to the structural analogues benzoic acid and isonicotinic acid. This sensor was also used to detect the nicotinamide (which is the amide of NA) in a Wahaha soft drink [309].

An odor sensor was developed by covering piezoelectric quartz crystals with nylon film supporting a polymer layer imprinted with 2-methylisoborneol. The device was used to detect off/flavour compounds which are produced from microorganism and are responsible of odor problems in drinking water and fish [310].

Another class of compounds detected by means of a MIM-based sensor are oxygenated terpene hydrocarbons. An imprinted membrane containing methactylic acid as functional monomer was polymerized on the surface of QCM sensor. The sensing property was examined in gas phase for evaluate the sensor sensitivity and selectivity. The MIP-QCM sensor can detect terpene-containing gases. The snsing properties evaluated in the gas phase were strongly influenced by the cross-linker/functional monomer/template ratio [311].

Fireman-Shoresh *et al.* first reported the preparation of a sensor imprinted with an organometallic complex ferrocene derivative via the sol-gel imprinting process [312]. The same technique was used to prepare metallothionein nanocrystalline titania film imprinted membranes on a QCM sensor. Metallothionein (MT) is a family of cysteine-rich proteins and is capable of binding metals and heavy metals. Owing to this property, MT is used as biomarker of metal contamination in food. The MT-imprinted membranes were used to cover a QCM sensor which was employed for monitoring the adsorption of the template and other proteins like bovine serum albumin (BSA) and bilirubin (BL). The adsorption capacity of MT, BSA and BL on imprinted membrane was 111.0 (mg·g^−1^) 43.0 (mg·g^‐1^), and 32.1 (mg·g^−1^), respectively. The results indicate that the imprinting process allowed the creation of a microenvironment based on shape selection and position that specifically recognizes template molecules. The adsorption observed for BSA and BL was due to non-specific interactions [312]. In fact, it was similar to that observed when using non-imprinted membranes, which were prepared using the same method but in absence of template [313].

Nitrocellulose poly(vinyl alcohol)-ionic-imprinted membrane (NCM-PVA-I-I) was prepared using Cu^2+^ as the template aiming at the determination of traces of this metal. When the template interacted with MIM it formed an ionic association with the fluoroscein anion allowing the emission of phosphorescence outside the recognition sites by electrostatic effects. The signal was proportional to the content of Cu^2+^. This novel sensing system was successfully used to detect copper traces in human hair and tea samples, proving highly selectivity [314].

## 6. Conclusions

The production of materials exhibiting biomimetic properties was one of the most important challenges of many researchers during the last decades. A considerable boost in this direction was given to the development of the molecular imprinting technology. In fact, since their invention imprinted materials were employed as elements capable of imitating the recognition aptitude of biological systems towards specific ligands. Owing to their high sensitivity, selectivity and stability, imprinted materials were successfully employed in different areas like separation, assay and catalysis. In particular, the requirement of novel testing approaches in substitution of the classical bio-recognition elements has determined a rapid development of materials in biosensor technology. Classical biorecognition materials are based on the use of biological molecules such as antibodies, enzymes, microorganisms, as recognition elements. Although these biological receptors are highly specific and sensitive, they suffer the disadvantages of being fragile and expensive. In addition, they possess low density of recognition sites; their regeneration as well as their storage is not easy and restricted. In addition, they suffer from availability, environmental intolerance and reproducibility issues. Biosensing systems using molecularly imprinted materials as recognition elements instead of biomolecules offer the possibility of overcoming some of these problems owing to their potential facility. The direct and rapid determination of an interaction between the recognition element and the template represents an encouraging factor for the development of such systems. This is because they do not need the addition of secondary reagents and the separation of free and bound reactants.

Traditional application of imprinted materials in sensor technology involved the use of imprinted polymer particles. However, the interface adhesion between the particles and the transducer surface can be weak. In addition, the response time is extremely long, owing to the slow mass transfer. Therefore, as an alternative to imprinted polymers, special formats of them like thin films or molecularly imprinted membranes are also under development. They are usually prepared by UV irradiation, thermal polymerization and via phase inversion techniques. MIMs-based sensors take advantage of their high selectivity and stability, which enable their long-term operation under conditions not tolerated by biomolecules without losing sensitivity.

Up to now MIMs-based sensors were developed for sensing water contaminants, drugs, food additives and some molecules in biological fluids. Despite the great application potential of these materials in sensor technology, we are aware that many efforts still need to be made to produce recognition systems optimized for each analyte. As an example, to simplify the imprinting of larger biological molecules there is a need for materials with more flexibility and a higher degree of accessibility. A great challenge for research in this field is the production of membranes imprinted with virus, which could be used in diagnosis and therapeutic treatments. The future of research in this area will have a thriving development benefitting from the integration of different disciplines. A contribution in this direction is to be found in the application of molecular modeling and combinatorial chemistry, which offer the possibility of optimizing the choice of template-functional monomer couples and to be able to predict the actions of the imprinted material at a molecular level. The literature data encourage the authors to believe that the different contribution offered by scientists in this field will have real importance in health care, environmental protection and industrial development.
